# A novel surgical procedure for bridging of massive bone defects

**DOI:** 10.1186/1477-7819-3-7

**Published:** 2005-02-03

**Authors:** Ulf R Knothe, Dempsey S Springfield

**Affiliations:** 1 Department of Orthopedic Surgery and Orthopedic Research Center, The Cleveland Clinic Foundation, Cleveland, OH, USA; 2 Department of Orthopedic Surgery, The Mount Sinai School of Medicine, New York, NY, USA

## Abstract

**Background:**

Bony defects arising from tumor resection or debridement after infection, non-union or trauma present a challenging problem to orthopedic surgeons, as well as patients due to compliance issues. Current treatment options are time intensive, require more than one operation and are associated with high rate of complications. For this reason, we developed a new surgical procedure to bridge a massive long bone defect.

**Methods:**

To bridge the gap, an *in situ *periosteal sleeve is elevated circumferentially off of healthy diaphyseal bone adjacent to the bone defect. Then, the adjacent bone is osteotomized and the transport segment is moved along an intramedullary nail, out of the periosteal sleeve and into the original diaphyseal defect, where it is docked. Vascularity is maintained through retention of the soft tissue attachments to the *in situ *periosteal sleeve. In addition, periosteal osteogenesis can be augmented through utilization of cancellous bone graft or *in situ *cortical bone adherent to the periosteal sleeve.

**Results:**

The proposed procedure is novel in that it exploits the osteogenic potential of the periosteum by replacing the defect arising from resection of tissue out of a pathological area with a defect in a healthy area of tissue, through transport of the adjacent bone segment. Furthermore, the proposed procedure has several advantages over the current standard of care including ease of implementation, rapid patient mobilization, and no need for specialized implants (intramedullary nails are standard inventory for surgical oncology and trauma departments) or costly orthobiologics.

**Conclusions:**

The proposed procedure offers a viable and potentially preferable alternative to the current standard treatment modalities, particularly in areas of the world where few surgeons are trained for procedures such as distraction osteogenesis (*e.g. *the Ilizarov procedure) as well as areas of the world where surgeons have little access to expensive, complex devices and orthobiologics.

## Background

Replacement of bone where there is none is one of the most challenging problems facing orthopedic surgeons today. In the case of tumor resection or trauma, massive bone defects must be filled with regenerate bone as quickly as possible in order to restore function. Current standards for bridging of massive bone defects in long bones generally follow a theme of *i*) filling the defect with bone autograft or allograft (including cancellous bone graft or bone transplantation via vascularized or non-vascularized fibula transfer) and *ii*) accelerating functional remodeling and integration through addition of physical and/or chemical stimuli such as tension (e.g. Ilizarov technique, which is a standard surgical treatment modality for bone transport whereby an osteotomy is performed far from the defect site and the transport segment thus created is moved, approximately one millimeter per day, under the constant tension by wires attached to a cumbersome external fixator, until the defect is bridged and the segment can be docked onto the other side of the defect), and orthobiologics (*e.g. *bone graft or bone graft replacement, BMP's). Surgical treatment modalities involving auto/allografting and bone regeneration via distraction osteogenesis are complex, time intensive procedures of inherently high risk due to vagaries of organ donation, in the case of allografts, and the complexity of soft and hard tissue salvage during the process of distraction osteogenesis. In addition, orthobiologics are costly and their dosage regimes as well as efficacy are currently the subject of much research [1-3].

Both bone grafting and bone transport procedures are complex for the surgeon as well as for the patient. Furthermore, they are susceptible to complications such as delayed union, extensive treatment time periods, infections, and insufficient mechanical function outcomes that can result in fractures. The high complication rates of these procedures exacerbate the previously mentioned difficulties associated with these treatment modalities, from the perspective of the surgeon as well as that of the patient. In short, the inherent risk of complications increases the need for patient compliance and clinical follow-up. Despite the effort associated with these procedures, their results are often less than satisfactory. Hence, the complexity and shortcomings of current state-of-the-art surgical procedures have provided impetus to develop a new treatment modality that provides a relatively straightforward, single step procedure with a high probability of success for the bridging of massive bone defects in long bones. The procedure is straightforward and can be implemented in operating rooms across the world without the need for high-tech equipment or expensive orthobiologics. The purpose of this manuscript is to describe the novel procedure.

## Technical innovation – methodology and proof of feasibility

**Figure 1:**
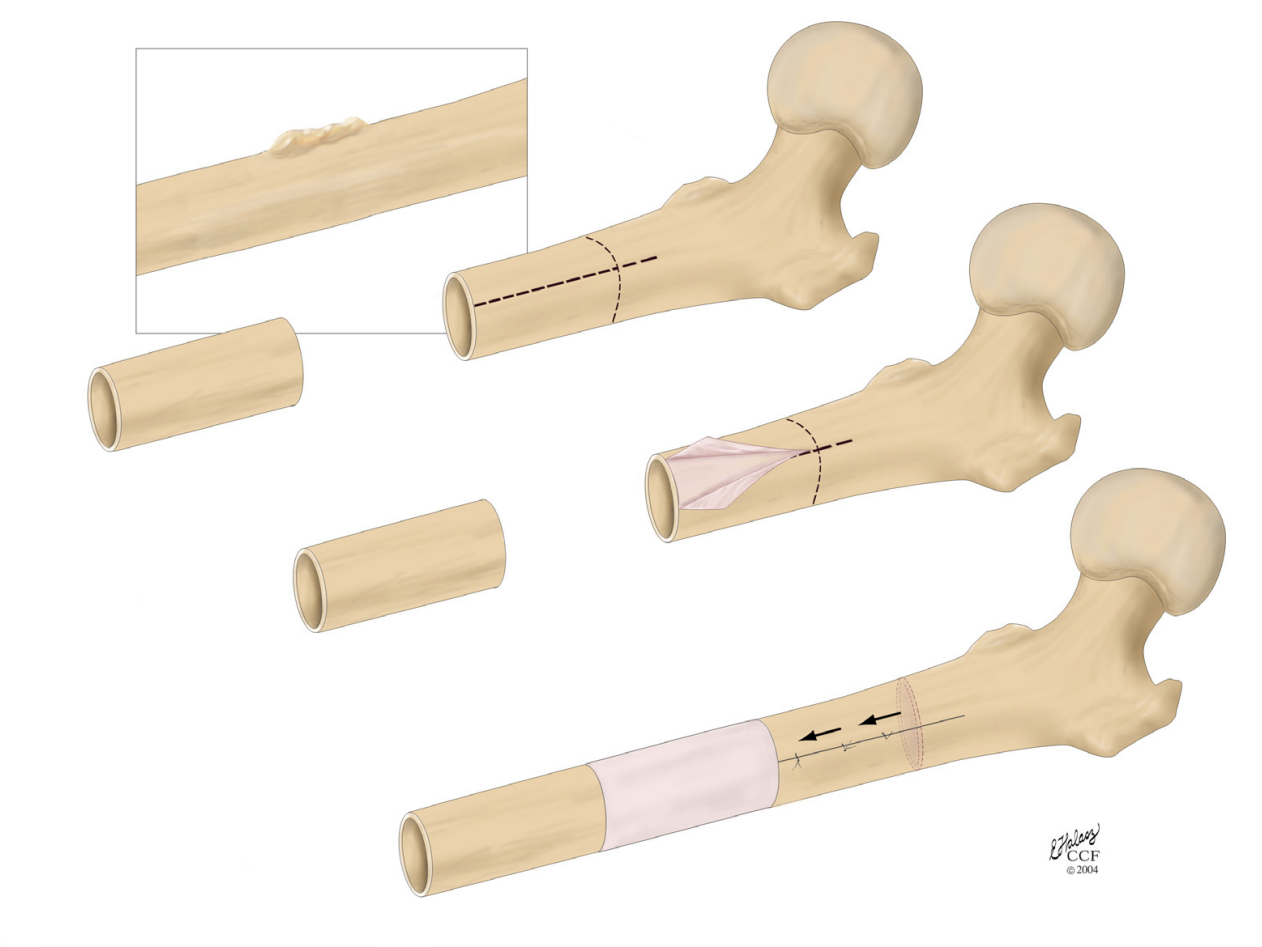
Schematic diagram showing the concept for the new surgical procedure.

The proposed procedure is applicable for clinical scenarios including tumor resection as well as debridement after an infection or non-union. In the case of reconstruction after tumor resection (Fig. 1A), a transport-segment of the diaphysis adjacent to the defect is pealed out of the surrounding periosteum (Fig 1B,C), an osteotomy is performed and the transport-segment is moved out of the periosteal sleeve and docked to the other side of the defect (Fig. 1D). The periosteal sleeve is then closed like a tube surrounding the newly created defect. Either an internal fixation device such as an intramedullary nail, a plate or an internal fixator or an external fixator can be used to provide stabilization through the healing and regeneration phase.

The proposed procedure depends to a large degree on bone's inherent healing strategies. Bone is a remarkably resilient tissue capable of adaptation to the most extreme biological and mechanical environments; this capacity for self-regeneration without scarring is based on bone's endogenous healing strategies. First, bone remodels itself through osteoclastic resorption and osteoblastic matrix apposition; by constantly reweaving itself, the structure is dynamic and optimal for prevailing mechanical function. Furthermore, the natural healing cascade of bone after trauma recapitulates embryonic endochondral ossification. Hence, modeling, growth and remodeling confer a means to regenerate functional tissue at any time in the life cycle of a bone. The "raw materials" necessary to replace bone are located in the environment or produced by the cells that do the work of regeneration, i.e. osteoclasts and osteoblasts. In the case of regeneration of bone in defects, further potentially key constituents to the formation of a functional regenerate in situ include a patent blood supply, chemical gradients of morphogens and/or cytokines, a template onto which the cells can anchor themselves during the rebuilding process (e.g. graft or a scaffold), and biophysical stimuli such as fluid flow and/or cell level strains. The proposed procedure essentially replaces the defect site in a pathological zone with a defect site in a healthy bed of tissue and provides for progenitor cells through the surrounding, healthy periosteum as well as many of the other key constituents for successful tissue regeneration, as defined above.

**Figure 2:**
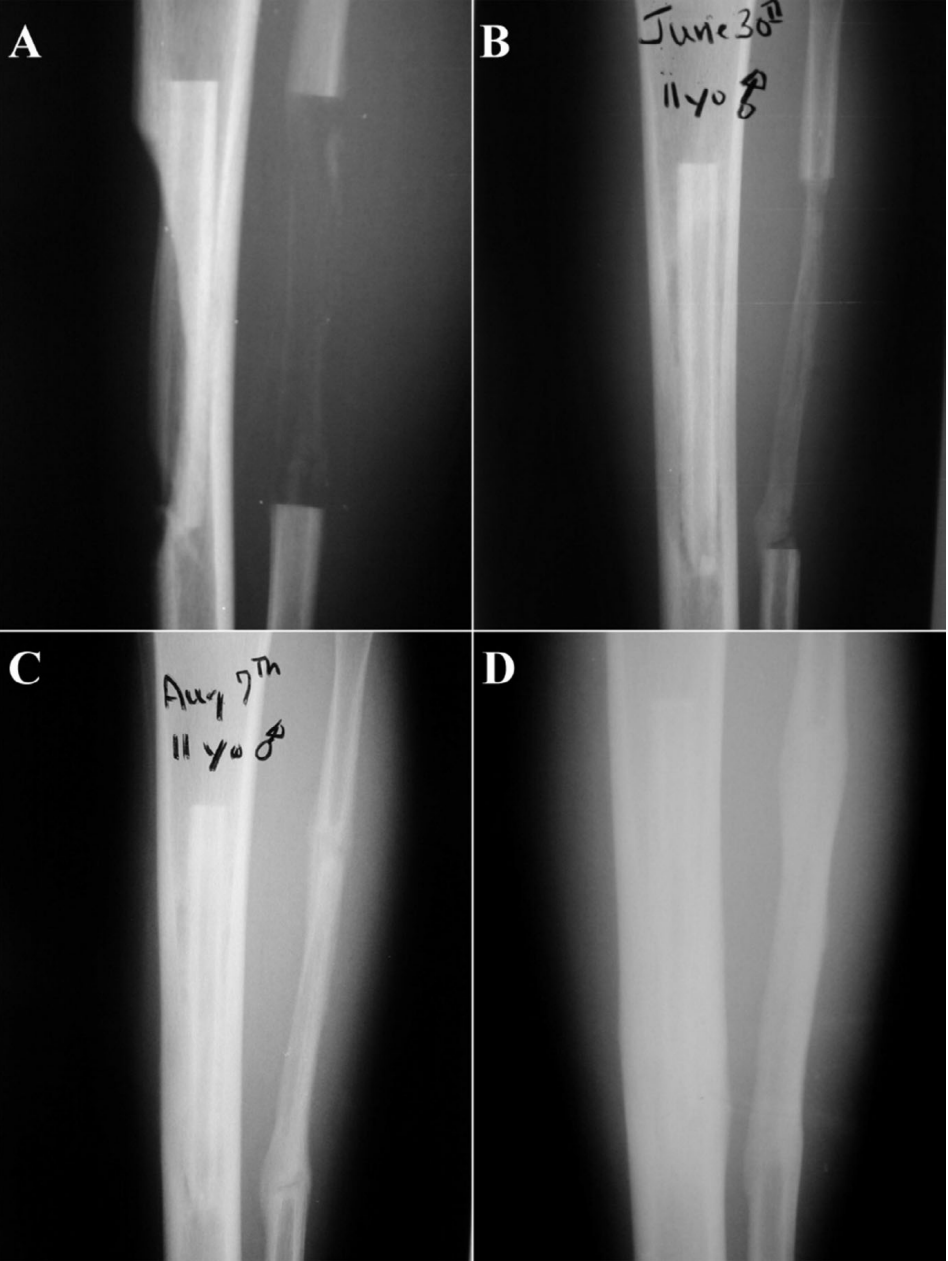
11 year male with malignant tumor. Fibula-pro-tibia following local resection. Cortical regeneration from periosteum. Performed at Mt. Sinai Medical Center, NYC, 2000. **A**: 3 weeks post-operative radiograph, **B**: 6 weeks post-operative, **C**: 3 months post-operative, **D**: approximately 6 months post-operative.

A clinical case described below demonstrates the osteoinductive potential of the periosteum and serves is a *proof of feasibility *for the proposed procedure. An 11-year old male presented with a low grade surface osteosarcoma of the tibia. After resection of the tumor, the fibula was resected for transfer and the surrounding periosteum was left behind to serve as an osteo-inductive and -conductive sleeve (Figure 2A). Already 3 weeks after the procedure, bone regenerate is visible within this sleeve (Figure 2A). Impressive remodeling of the fibula is also evident in follow up radiographs and includes extensive remodeling of the intramedullary canal by three months post procedure (Figure 2B and 2C). Based on this clinical case as well as one author's previous experience with an *in vivo *segmental defect in an ovine model [4,5], the osteogenic potential of the periosteum as a source of progenitor cells and as a "membrane" or boundary template for guided bone generation is demonstrated. Taking this one step further, the corresponding author conceived of the idea to exploit the potential of the healthy periosteum by moving the defect site from a pathological zone to a healthy one and then providing sufficient mechanical stability to let bone's endogenous healing capacity regenerate functional tissue within the new defect zone.

## Discussion

The proposed procedure is novel in that it introduces for the first time the possibility to bridge a massive defect in a long bone using a single stage procedure. Furthermore, the proposed procedure has several advantages over the current standard of care including ease of implementation, lack of requirement for specialized implants (intramedullary nails are standard inventory for surgical oncology and trauma departments) or costly orthobiologics, and rapid patient mobilization. This makes the proposed procedure a viable and potentially preferable alternative to the current standard treatment modalities, particularly areas of the world where few surgeons are trained for procedures such as distraction osteogenesis (*e.g. *the Ilizarov procedure) as well as where surgeons have less access to expensive, complex devices and orthobiologics.

## Conclusion

In summary, the authors propose a new procedure which obviates the need for several surgical procedures, reduces the risk for complications, reduces the time frame for the treatment and is much more comfortable for and requires less compliance of the patient. This novel, one stage procedure exploits the osteogenetic potential of the periosteum for bone formation to bridge the defect with concomitant bone transport and does not require the use of expensive hardware or orthobiologics.

## Competing interests

The author(s) declare that they have no competing interests.

## Authors' contributions

**UK **conceived of the technical innovation, organized its design and coordination, and drafted the manuscript. **DSS **was the senior surgeon in the clinical case showing feasibility. Both authors read and approved the final manuscript.
